# Transition to professional practice: Perspectives of new nursing graduates of Nepal

**DOI:** 10.1186/s12912-023-01418-2

**Published:** 2023-08-18

**Authors:** Sital Gautam, Anju Poudel, Kalpana Paudyal, Mangal Maya Prajapati

**Affiliations:** 1 School of Nursing & Midwifery, Patan Academy of Health Sciences, Lalitpur, Nepal; 2 Hospital Nursing Administrator, Health Directorate, Gandaki Province, Nepal; 3 Nepalgunj Nursing Campus, Tribhuvan University, Institute of Medicine, Nepalgunj, Nepal; 4 Acting Nursing Director, Civil Service Hospital, Kathmandu, Nepal

**Keywords:** New nursing graduates, Professional practice, Transition

## Abstract

**Background:**

Internationally, the transition from student nurse to practicing nurse is recognized as being the most stressful period. Yet very little is known about how new nursing graduates perceive this transition in Nepal. The study aimed to explore new nursing graduates’ perceptions of the transition to professional practice.

**Methods:**

A qualitative descriptive methodology was used. In-depth semi-structured interviews were conducted with 10 purposively recruited participants from two private hospitals in Nepal. Data were analyzed using inductive thematic analysis. The Consolidated Criteria for Reporting Qualitative Research (COREQ) guidelines were used to report the findings of this study.

**Results:**

New nursing graduates perceived the transition to professional practice as an intense experience. Inductive thematic analysis yielded four intrinsically linked themes that encompassed new nursing graduates’ transition experiences: ‘getting hit by reality’, ‘losing confidence’, ‘feeling unsupported’, and ‘gathering strengths.’ The theme ‘getting hit by reality’ included three sub-themes: ‘gap between theory and practice,’ ‘no protective shield,’ and ‘plethora of responsibilities’, which explains nurses’ initial encounter with real-world practice. The theme ‘losing confidence’ contained three sub-themes: ‘being fearful,’ ‘being ignored,’ and ‘being accused,’ which describes how nurses started losing confidence as they confronted the real side of the profession. The theme ‘feeling unsupported’ included two sub-themes: ‘left without guidance,’ and ‘limited support from seniors,’ which explains how nurses perceived their work environment. The theme ‘gathering strength’ contained two sub-themes: ‘reflecting’ and ‘asking for help,’ which describes how nurses coped with the challenges related to the transition.

**Conclusion:**

To facilitate the transition to practice, educational institutions must impart to students a realistic understanding of the transition process, address the theory-practice gap, and collaborate with hospitals. Similarly, hospitals should have realistic expectations from new nurses, assign work according to their capabilities, and allow them sufficient time for role integration. Likewise, well-conceived detailed orientation, mentorship or preceptorship programs, and regular professional development programs are vital to easing the transition. Furthermore, establishing and maintaining a supportive work culture, which promotes equity, respect, and safety among employees, is crucial for positive transition experiences.

**Supplementary Information:**

The online version contains supplementary material available at 10.1186/s12912-023-01418-2.

## Introduction

Globally, the healthcare system is experiencing a high nursing turnover. According to the National Health Care Retention & RN (Registered Nurse) Staffing Report in the United States, the average turnover rate for RN is 25.9% [1]. Evidence shows that one-fourth of new nursing graduates plan to leave their jobs within the first year of registration [2]. The turnover intention precedes the actual turnover [3]. This suggests a need for reflection on how new nursing graduates perceive the transition to clinical practice and how they can be supported during the early stages of their careers.

Internationally, the transition from student nurse to practicing nurse is recognized as being the most stressful period for nurses. Transition means “moving from one state to another one which is usually associated with significant changes in goals, roles, and responsibilities” [4]. In the case of new nursing graduates, a transition is a period of professional socialisation where they acquire knowledge, skills, and values of the nursing profession and culture [2]. Studies have shown that new nursing graduates face multiple problems during the transition period, mainly due to a lack of orientation, an unwelcoming environment, heightened responsibilities, disproportionate workload, unrealistic expectations, and horizontal violence. New nursing graduates also experience role ambiguity, overload, and stress [5–10]. In addition, reality and transition shock are significant issues for new nursing graduates. Here, reality shock denotes a mismatch between the expectation and reality of the role, and transition shock implies a conflict between universities’ instilled values and real-world nursing practice [6, 11]. Furthermore, common to the experience of new nursing graduates is an inadequate support system in the workplace [2, 12].

The multifaceted challenges associated with the transition can negatively impact new nursing graduates’ well-being. Indeed, previous studies have demonstrated that new nursing graduates experience anxiety, emotional exhaustion, fear, and frustration amidst transition [13]. Therefore, supporting new nursing graduates in their transition journey is crucial to ensure retention. Different interventions have been developed internationally to support new nursing graduates in their transition to clinical practice. These include, but are not limited to, internship, orientation, mentorship, and preceptorship [14, 15].

While considerable international literature exists on how nurses perceive transition and the ways to support them, it has not gained enough scholarly attention in Nepal. The context of Nepalese nursing education, practice, culture, and health system is different from western countries. Woo and Newman (2019) pointed out that perspectives on transition can vary according to the personal, professional, and organizational contexts of nurses [7]. Researchers, therefore, must incorporate the participants’ context while studying transition. In Nepal, only few studies have emphasized the perspectives of new nursing graduates on the topic of transition [16]. Moreover, the studies have only focused on the transition experiences of new nursing graduates within the first three months of their practice. To gain an in-depth understanding of the perspectives on transition, it is essential to include nurses who have worked in the hospitals for at most one year [2]. The current study addresses this gap by exploring new nursing graduates’ perceptions of the transition to professional practice during the first year of employment.

### Research aim

The study aims to explore new nursing graduates’ perceptions of the transition to professional practice.

## Methods

### Design

The study employed qualitative descriptive methodology embedded in the principles of naturalistic inquiry to explore new nursing graduates’ perceptions of the transition to professional practice. Descriptive approach “is especially amenable to obtaining straight and largely unadorned (i.e., minimally theorized or otherwise transformed or spun) answers” [2]. Naturalistic inquiry is a generic orientation to an inquiry that implies studying a phenomenon in its natural state. Consistent with the fundamental tenets of naturalistic inquiry, the present study began with no pre-existing commitment to any theoretical views regarding the phenomenon of interest [17, 18]. Accordingly, the phenomenon was explored in its natural state from the perspective of those experiencing it within their contexts. This methodology is indicated when there is a paucity of research on the topic, and straight descriptions about the phenomenon of interest are intended, as in the case of the present study. The chosen methodology also allowed staying closer to the data and participants’ words as intended in this study [17, 19]. The Consolidated Criteria for Reporting Qualitative Research (COREQ) guidelines were used to report the findings of this study [20].

### Participants

Participants were recruited from two private hospitals in Chitwan, Nepal using purposive sampling techniques. The hospitals were chosen because both were multi-specialty and private. Given the high nurse turnover rate in private hospitals, the chances of recruiting new nursing graduates were high. Hospital 1 was a 100-bedded hospital established in 2006. The total number of nurses was 47. The hospital maintained a 1:5 nurse-to-patient ratio. Similarly, there were 55 beds in Hospital 2, which was established in 2013. Hospital 2 had appointed 25 nurses and maintained a 1:4 nurse-to-patient ratio. Both hospitals had provisions for rotating nurses in different departments after a certain period. Orientation provisions for new nurses were brief and general in both hospitals. Nurses were eligible to participate if they were new nursing graduates and had worked in the hospitals for at most a year. In total, we recruited ten new nursing graduates.

### Data Collection

Data collection occurred from March 2021 to April 2022. The potential participants were approached via the hospitals, where they worked. The managers of the hospitals provided the list of new nursing graduates who had worked there for at most a year along with their contact numbers. The potential participants were contacted and informed about the study. Willing participants received an information sheet and a consent form. Participants decided on the interview dates and times. After receiving written informed consent, SG and AP collected data through in-depth semi-structured face-to-face interviews. Thirteen interviews were scheduled. The total number of interviews included was 10. The interview guide consisted of open-ended questions about participants’ perceptions of the transition to professional practice (Table 1). Follow-up questions elicited further information about specific areas of interest. The interview schedule was developed with the help of the literature review and approved by three experts, including a senior qualitative researcher, nursing staff, and nursing faculty. The interview guide was pilot tested in two new nursing graduates and modifications were made to the language and wordings. Interviews lasted from 35 to 42 mins. Interviews were digitally recorded. Field notes were taken after the interviews to document specific gestures, facial expressions, tone of voice, and context of the study, which helped in data analysis. Interviews were transcribed verbatim in the Nepali language and translated into English by SG. The English version of the interview transcripts was then translated back into the Nepali language by a professional bilingual translator. The original and back-translated interview transcripts were verified to ensure both versions implied the same meaning. Data collection stopped when new data fitted within the previously developed themes/subthemes and did not add anything significant to the analysis, also referred to as data saturation [17].

**Table 1:** Interview schedule

Interview schedule	
1. Can you please tell me how you perceived the transition to professional practice?	
2. What difference did you find while being a student versus a professional nurse?	
3. Please tell me about the most satisfying aspects of being a new nursing graduate.	
4. What kind of challenges do you face while working as a new nursing graduate?	
5. How did you adjust to those challenges, and what helped?	
6. Do you wish for any support or facilities that could make the transition experience better for new nursing graduates?	

### Ethical considerations

The Ethical Review Board (ERB) of Nepal Health Research Council (NHRC) provided ethical approval for the study (Reference number: 57/2021P). The selected hospitals provided written approval to conduct the study. The information sheets with study details were provided and informed consent was obtained before data collection. Participants were assured that participation in the study was voluntary, they could withdraw at any time, and non-participation would not impact them in any way. No power imbalances were identified as SG and AP, registered female nurses, were not practicing nursing in either of the hospitals. To ensure privacy, data were collected separately from each respondent in a quiet place. Only the research team had access to the data.

### Data analysis

Braun and Clarke’s reflexive thematic analysis was employed. Thematic analysis signifies “a method for identifying, analysing, and reporting patterns (themes) within data” [21]. The themes were data-driven, which implies an inductive approach to theme generation. We followed six steps of reflexive thematic analysis suggested by Braun and Clarke [21]. Firstly, the transcripts were read multiple times to familiarize with the data, and preliminary ideas were noted. Next, initial codes were generated across the entire data set. By collating those codes, themes and sub-themes were identified. Then, themes were reviewed relating them to the generated codes and the entire data set. Next, the themes were refined, named, and defined. Finally, a report was produced integrating compelling extracts [21]. SG and AP undertook the initial analysis. Then, the remaining authors discussed themes, discerned potentially inflicted codes, identified gaps, and reviewed accordingly.

### Rigor

To ensure rigor, we followed techniques suggested by Lincoln and Guba (1985) [18]. Peer review was conducted for credibility, and it was found that they could relate to most of the themes. Likewise, member-checking with the participants indicated that the findings resonated with their experience. Moreover, a negative case analysis was conducted for credibility. To ensure transferability, thick descriptions were provided, and substantial excerpts were integrated into the findings [22]. Detailed descriptions of research methods and decisions undertaken were provided for dependability. Similarly, SG and AP made coding and analytical decisions, maintained a log of decisions, and a reflexive journal throughout the research process for confirmability. The remaining authors regularly reviewed analytic documents to identify potential assumptions and reflect on participants’ true concerns. The research team worked together to reach a consensus on the interpretation of the data [18].

## Findings

Ten new nursing graduates aged 22–24 years (Median age: 23years) participated in the study. All participants were female, unmarried, and practiced the Hindu religion. Participants had completed their bachelor’s degree in nursing (BSc. Nursing) from private institutes and worked in the hospitals in Chitwan for 4 to 12 months (Median duration: 8.5 months). New nursing graduates perceived the transition to professional practice as an intense experience. Thematic analysis yielded four intrinsically linked themes that encompassed new nursing graduates’ transition experiences: getting hit by reality, losing confidence, feeling unsupported, and gathering strengths (Table 2).

**Table 2:** Themes, subthemes, codes

Themes	Subthemes	Codes
Getting hit by reality	Gap between theory and practice	Maximum theory while studying
		Minimum practical exposure Ample amount of work in the ward Different practice
	No protective shield	No one to guide/protect
	Plethora of responsibilities	Heavy workload
		Full responsibility
Losing confidence	Being fearful	Fear of procedures
		Fear of making mistakes Fear of getting scolded Anxiety Nervousness Need for verification
	Being ignored	Working as instructed
		Not knowing rationale Not being able to answer queries raised Being mistrusted Being bypassed
	Being accused	Easy target
		False accusation Guilt transfers
Feeling unsupported	Left without guidance	Left alone
		No detailed orientation
		No training
	Limited support from seniors	Being controlled
		Being overburdened Being troubled Being dominated Being ordered Being undermined Being scolded Being treated differently Wanting to leave Some supportive seniors
Gathering strength	Reflecting	Evaluate own work
		Search Read Watch Improve
	Asking for help	Ask seniors Request for guidance

### Getting hit by reality

The theme ‘getting hit by reality’ includes three sub-themes: ‘gap between theory and practice,’ ‘no protective shield,’ and ‘plethora of responsibilities’, which explains nurses’ initial encounter with real-world practice.

### Gap between theory and practice

Before commencing employment, the nurses reported being excited about the completion of educational preparation and beginning a new job. However, following a few months of work, nurses felt that the real-world practice was unlike what they anticipated. Many nurses reported a gap between theory and practice. Nurses described that actual nursing practice was an ‘ample amount of work’ or ‘ocean of work,’ which required ‘minimum’ theoretical knowledge. In nursing college, however, nurses were prepared more theoretically than clinically. A nurse explained, *“I attended a lot of theory classes … but I did not get extensive clinical exposure when I was a student”* (Participant 1, Intensive Care Unit (ICU). As students, nurses were not permitted to perform several nursing procedures. Also, what they learned in nursing college and what was practised in the hospital differed significantly.

Another nurse stated, *“There was a marked difference between what I was taught in theory vs. how it was carried out clinically [referring to procedures]”* (Participant 3, ICU). A new nursing graduate who had completed six months in the hospital, recounted her experience of getting hit by reality:

*While studying, there were lots of theory classes to attend but the practical exposure was minimum … When I was a student, I knew nothing about real-world practice … We were not allowed to perform many procedures [in the hospital]. So, I did not learn much … After commencing employment, there was an ample amount of work to be finished and theoretical knowledge had a minimal role in it ... That is why I became hopeless.* [Participant 4, Medical Ward]

This quote demonstrates that she felt her education had not prepared her for the roles and responsibilities in the real world. Eventually, it led to her feeling hopeless.

### No protective shield

Some nurses expressed that, unlike their expectations, the real-world practice was a lonely journey with no protective shield. As students, nurses were accompanied and guided by teachers, clinical supervisors, or staff, which led to the feeling of being protected. However, as soon as they became staff, they no longer had that protective shield and, hence, experienced loneliness. A nurse stated, *“While we were students, we were not assigned a fair share of responsibility … and we had teachers who would guide us … But when I started working, I immediately became solely responsible for too many things”* (Participant 10, Emergency Ward).

### Plethora of responsibilities

Nurses were only entrusted with a small number of responsibilities when they were students. However, a few months into employment, the nurses encountered a plethora of responsibilities. A nurse remarked, *“While studying, I used to be assigned two or three patients. In practice, a staff member is given full responsibility for five to seven patients while there will be only two or three staff working. Consequently, the workload is heavy”* (Participant 4, Medical Ward). For nurses, dealing with numerous responsibilities in a brief period was overwhelming.

### Losing confidence

The theme ‘losing confidence’ contained three sub-themes: ‘being fearful,’ ‘being ignored,’ and ‘being accused,’ which describes how nurses started losing confidence as they confronted the real side of the profession.

### Being fearful

Encountering real-world practice made nurses fearful. Many nurses reported being ‘nervous’, ‘frightened’, or ‘anxious’ before undertaking any major or minor procedures in the initial days. Nurses developed a fear of ‘making mistakes’ and ‘getting scolded’. Consequently, a growing need for validation unfolded, wherein new nurses could not perform any procedures without verifying with their seniors. A nurse recalled being fearful in her initial days:

*I was terrified in the initial days ... I used to verify 2–3 times [with seniors] before commencing any procedures. Although I had some knowledge of the procedures, I used to ask to confirm, be sure, and be confident. I was afraid. I felt my confidence was at a low point and it was uncomfortable asking the same thing [referring to procedures] multiple times.* [Participant 2, Emergency Ward]

Another nurse confirmed, *“In the beginning, I was frightened of the working environment and people. [I was afraid] that [I] will be scolded if I made mistakes”* (Participant 6, Medical Ward).

### Being ignored

Some nurses confronted that due to the lack of confidence in the beginning, they only followed seniors’ instructions and worked as directed without understanding the underlying rationale. It was also difficult for nurses to answer patients’ and visitors’ queries when they were new. Therefore, nurses were often ignored by patients and visitors. A nurse expressed:

*When I was new, I could not give rationales confidently. I did not know why I was doing certain things [referring to nursing interventions] … For instance, when visitors used to ask about the investigations, I did not have any idea why it was being done. I did it because the doctor recommended doing so ... When that happened, visitors noticed that I was a new nurse and started ignoring me … People think that new nurses know nothing, and they do not trust us.* [Participant 8, Surgical Ward]

She also confirmed that mistrust and ignorance from patients and visitors ‘further reduced’ her level of confidence.

### Being accused

When nurses lacked confidence in their work, they also became easy targets for false accusations and guilt transfer. For instance, a new nursing graduate shared one incident when she was falsely accused by senior nurses:

*It happens when you are a new nurse … In the hospital, there was one cardiac patient with an allergy. The doctor ordered medicine A and B. I told them he was a cardiac patient and suffered a heart attack in the past … Even then, the doctor insisted to give that medicine and said nothing will happen ... The senior sister who had worked there for 6 years gave that medicine to the patient … The patient went on asystole ... Then, that senior sister blamed me. I did nothing. I could not say a word at that time ... [I] had to work with them ... It is dangerous when you are a new nurse.* [Participant 1, ICU]

She confirmed that these kinds of incidents further ‘jeopardized’ her confidence and ‘demotivated’ her.

### Feeling unsupported

The theme ‘feeling unsupported’ included two sub-themes: ‘left without guidance,’ and ‘limited support from seniors,’ which explains how nurses perceived their work environment.

### Left without guidance

Feeling unsupported was common in the experiences of many nurses. Most nurses revealed that they were left alone in the very beginning without ‘detailed orientation’ and ‘training’, which led to errors in work and many negative experiences. One new nursing graduate explained how she was left without guidance:

*I did not receive a detailed orientation. In the beginning, new nurses are usually posted alongside senior staff. In my case, that did not happen … [One day], intubation was going on and I did not know where an equipment was placed. I was new and they (other staff) had the practice of cutting the catheter of the suction tube, which I did not know … I gave it (suction tube) as it is, and the doctor told me to leave the job that very moment … [That is my] worst experience till now.* [Participant 3, ICU]

Another nurse conveyed, *“It was strenuous to work straight away without any orientation”* (Participant 5, Surgical Ward).

Nurses expressed that in-depth orientation is central to effective transition. Nurses also reported that they got no opportunity to participate in conferences, workshops, seminars, or specialized training, *“I got no chance to get involved in orientation, training, seminar or discussions”* (Participant 7, Medical Ward). Similarly, another nurse stated, *“As a student, you do not get a chance to learn all that is needed in the workplace. The workplace should arrange for discussion classes, orientation, training, and seminars”* (Participant 5, Surgical Ward).

### Limited support from seniors

Some nurses reported that they received limited support from their seniors. Nurses expected seniors to be ‘supportive’, ‘kind’, ‘cooperative’, ‘polite’, ‘caring’, and ‘helpful’. Unlike their expectation, nurses reported being ‘controlled’, ‘overburdened’, ‘troubled’, and ‘dominated’ by seniors. For instance, a nurse described:

*Seniors overburdened us with work beyond our capacities. They said, ‘you are juniors,’ and ordered us to do all kinds of work. I was given tremendous responsibilities and accountabilities. In addition, [there were] so many rules and regulations to follow … We had to help the seniors even after completing our assigned beds ... Seniors used to complain even when we were 5 minutes late to work ... They sent us to other wards when our ward was free .... They troubled us a lot and they did not grant leave when I was sick. Adapting to this environment was a challenge. One of my friends faced a similar situation and she left her job within 2 weeks.* [Participant 6, Medical Ward]

Another nurse agreed, *“Seniors treat juniors differently. I felt bad”* (Participant 7, Medical Ward).

Other nurses also expressed that seniors ‘ordered,’ ‘scolded,’ and ‘undermined’ them when new. Feeling unsupported by the seniors made the nurses’ adjustment more challenging. For a few nurses, lack of support from the seniors also became the main reason to leave the job, *“Due to such behaviours, nurses tend to leave [the job]”* (Participant 2, Emergency Ward).

Only two new nursing graduates concluded that seniors’ support and encouragement eased their transition process, *“Seniors built my confidence and taught me how to work* (Participant 4, Medical Ward),*” “The transition wasn’t very difficult because the seniors were supportive”* (Participant 5, Surgical Ward). Nurses reported a need for healthy interaction, a supportive environment, and collegial relationships in the workplace.

### Gathering strength

The theme ‘gathering strength’ contained two sub-themes: ‘reflecting’ and ‘asking for help,’ which describes how nurses coped with the challenges related to the transition. The way of gathering strength differed among nurses.

### Reflecting

Some nurses started reflecting on their practice, which meant they began to analyze their work, and think about ways to improve it. Nurses started searching, reading books, and watching videos about cases and topics that confused them. Similarly, nurses began to read more about the procedures in which they made mistakes. A nurse, who had completed eight months in the hospital, shared how she strategized to gather strength:

*Whenever I made mistakes [in the ward], I used to go back, watch the procedure on YouTube, and re-read the [related] theory. I experienced that we need to be aware of a lot of other things than theory alone. That is why when new cases came, I started searching about those cases rather than learning them from the doctor.* [Participant 8, Surgical Ward]

As analyzed in the excerpt, she started evaluating her own work and educating herself to improve her knowledge and practice. Another nurse stated, *“I started becoming alert before doing work to make sure that I make no mistakes”* (Participant 3, ICU).

### Asking for help

A few nurses asked for help to develop and advance knowledge and skills, *“Seniors answered my queries, corrected my understanding, and guided me when I made mistakes”* (Participant 2, Emergency Ward). Over time, the above-mentioned strategies improved the nurses’ knowledge and skills in the core areas of practice, helped them in rational decision-making, and boosted their confidence. Their learning gained in the process became their major source of strength to move forward, *“I learned new things every day. I felt I was improving day by day. I felt proud, confident, and satisfied”* (Participant 10, Emergency Ward). Better appreciation from patients also gave strength to nurses.

## Discussion

This research aimed to explore new nursing graduates’ perceptions of the transition to professional practice. Overall, new nursing graduates perceived transition as an intense experience. This finding resonates with most previous studies in this area, revealing that the transition is a stressful experience for new nurses [7, 23]. An important finding in this study was that new nursing graduates felt unprepared for the new role. Nurses identified that the job demanded hands-on experience, however, they were prepared more theoretically than practically. These results corroborate the findings of previous studies [7, 23, 24]. The feeling of inadequacy among new nurses is often linked with workplace expectations. In clinical practice, new nursing graduates are often expected to be job or workplace ready. Nevertheless, new nursing graduates are still learning how to apply their theoretical knowledge, consolidate their clinical skills, think critically, and make comprehensive clinical decisions. Similarly, they are still figuring out how to comprehend professional responsibilities, and frontiers of ethical practice [25]. Therefore, it is unrealistic to expect new nursing graduates to be job-ready immediately after joining. As newcomers, nurses should be given sufficient time to be well-versed in their roles [17].

Another important finding of the study was that the new nursing graduates experienced huge differences between what was taught and practiced. The gap between theory and practice is commonly reported by new nursing graduates throughout the world [2]. These experiences have been described as a part of reality shock, which means realizing that professional reality does not necessarily meet the expectation [18]. These findings indicate the need for educational institutions to impart a realistic understanding of the real-world practice. In addition, educational institutions should identify gaps in theory and practice, address them, and keep student nurses updated. Close collaboration between educational institutions and hospitals is also pivotal in addressing this gap [12].

The current study also found that a sudden heightened responsibilities and accountabilities intimidated new nursing graduates. Similar to the findings of the current study, Woo and Newman identified that it is daunting for new nurses to shift from a sheltered student life with limited responsibilities to unguarded professional life full of vast responsibilities [7]. Therefore, it is recommended to reduce the workload of new nursing graduates in the first few weeks to allow them time to adjust to a new role. Furthermore, new nursing graduates should be assigned responsibilities according to their capabilities and provided support as needed [26]. The gradual integration of new nursing graduates into their professional role is essential.

Another key finding of this study was that new nursing graduates were often ignored and untrusted by patients and visitors. Furthermore, new nursing graduates encountered false allegations from seniors, which further reduced their confidence and demotivated them to work. A study conducted in Singapore also found that new nurses, in most instances, are stigmatized because of the knowledge and practice deficit. Similarly, the study identified that being stigmatized heightens tension and damages the confidence of new nurses [7]. However, the experience of being falsely accused by seniors was uniquely reported by Nepalese nurses. This finding highlights the need for promoting equity, respect, and employee safety in the workplace to ease the transition.

A significant finding of this study was that new nursing graduates were deprived of detailed orientation, which made their transition experience strenuous. This result mirrors that of a past study, which identified that in many cases, new nursing graduates are ‘left by themselves’ with ‘no orientation’ [23]. When new nurses are ‘thrown in the deep end,’ meaning alone without any orientation, it makes their transition experience stressful [9]. These findings have important implications for establishing properly conceived orientation programs for assisting new nursing graduates in their transition journey. Similarly, a mentorship or preceptorship program is central to smooth transition [27]. In mentorship or preceptorship programs, qualified and experienced nurses are assigned to guide new nursing graduates, which can help them to gain insight into real-world practice, be confident, and adapt [2].

Another key finding was that new nursing graduates in this study felt unsupported by seniors. Most of them reported being ‘controlled,’ ‘overburdened,’ ‘troubled,’ ‘dominated,’ ‘ordered,’ ‘scolded,’ and ‘undermined’ by seniors. These results reflect those of Woo and Newman who identified ‘unsupportive, oppressive, abrasive cultures (horizontal violence)’ as a major source of stress for new nursing graduates [7]. The issue of an unsupportive environment challenging the transition experience of new nurses has also been highlighted by many other studies [23, 28]. Nurses in the current study explained that seniors exercising power against new nurses is part of a hierarchical culture, which is deeply entrenched in the nursing profession in Nepal. This finding highlights the importance of establishing a supportive culture in the nursing profession to facilitate the transition. Previous studies also uphold that a supportive environment is central to a positive transition experience [23, 27].

The most striking finding of this study is that despite the intense transition experiences, most new nursing graduates gathered strengths by being self-reflective and asking for help. This finding is consistent with the grounded theory of transition developed by Duchscher [11]. Duchscher identified that after difficult initial months, new nurses start searching for rationales which leads to marked advancement in their knowledge and skill [11]. Therefore, hospitals should create an effective learning environment for new nurses and arrange for continuous learning opportunities such as conferences, seminars, training, and workshops [16, 27].

### Limitations

Although providing a unique perspective, the study is limited to the group of new nursing graduates recruited from the selected hospitals in Nepal. Likewise, the study is limited to the unique health care system and cultural context of nursing practice in Nepal. Similarly, the type of institutes from which these participants graduated and where they practiced during their courses might have affected their transition experiences. Also, the differences in the context of the selected hospitals might have influenced the development of perceptions differently. However, the findings, as mentioned above, fit with many hospitals; thus, the perceptions may well be transferable to hospitals in other countries. Future research involving a bigger group of new nursing graduates from diverse hospitals is needed to add variation to the transition experiences. Moreover, participant observation could not be conducted, which could add richness to the transition experiences.

## Conclusion

The study provides unique information on how new nursing graduates in Nepal perceive the transition to professional practice. Transition to professional practice is a critical period for nurses which necessitates support. To facilitate the transition, educational institutions must impart to students a realistic understanding of the transition process, address the theory-practice gap, and collaborate with hospitals. Similarly, hospitals should have realistic expectations from new nurses, assign work according to their capabilities, and allow them sufficient time for role integration. Likewise, well-conceived detailed orientation, mentorship or preceptorship programs, and regular professional development programs are vital to easing the transition. Furthermore, establishing and maintaining a supportive work culture, which promotes equity, respect, and safety among employees, is crucial for positive transition experiences. The findings of this study will be beneficial for nurses, nursing educators, and managers to recognize areas of improvement in current practice and design effective interventions according to identified needs.

### Electronic supplementary material

Below is the link to the electronic supplementary material.

Supplementary Material 1

## Data Availability

The datasets used and/or analysed during the current study are available from the corresponding author upon reasonable request.

## References

[1] NSI nursing solutions. NSI National Health Care Retention & RN Staffing Report. 2022. Available from NSI_National_Health_Care_Retention_Report.pdf (nsinursingsolutions.com).

[2] Kreedi F, Brown M, Marsh L, Rogers K (2021). Newly Graduate Registered Nurses’ Experiences of transition to clinical practice: a systematic review. Am J Nurs.

[3] Lee J (2022). Nursing home nurses’ turnover intention: a systematic review. Nurs Open.

[4] Azimian J, Negarandeh R, Fakhr-Movahedi A. (2014). Factors affecting nurses’ coping with transition: an exploratory qualitative study. Glob J Health Sci. 2014; *6*(6): 88–95. 10.5539/gjhs.v6n6p88 PMC4825481 25363117

[5] Reebals C, Wood T, Markaki A (2022). Transition to practice for new nurse graduates: barriers and mitigating strategies. West J Nurs Res.

[6] Aldosari N, Pryjmachuk S, Cooke H (2021). Newly qualified nurses’ transition from learning to doing: a scoping review. Int J Nurs Stud.

[7] Woo MW, Newman SA (2020). The experience of transition from nursing students to newly graduated registered nurses in Singapore. Int J Nurs Sci.

[8] Rosi IM, Contiguglia A, Millama KR, Rancati S (2020). Newly graduated nurses’ experiences of horizontal violence. Nurs Ethics.

[9] Hussein R, Everett B, Ramjan LM, Hu W, Salamonson Y (2017). New graduate nurses’ experiences in a clinical specialty: a follow up study of newcomer perceptions of transitional support. BMC Nurs.

[10] Feddeh SA, Darawad MW (2020). Correlates to work-related stress of newly-graduated nurses in critical care units. Int J Caring Sci.

[11] Duchscher JEB. From surviving to thriving. 1st ed. Nursing the Future; 2012.

[12] Ankers MD, Barton CA, Parry YK (2017). A phenomenological exploration of graduate nurse transition to professional practice within a transition to practice program. Collegian.

[13] Graf AC, Jacob E, Twigg D, Nattabi B (2020). Contemporary nursing graduates’ transition to practice: a critical review of transition models. J Clin Nurs.

[14] Hampton KB, Smeltzer SC, Ross JG (2021). The transition from nursing student to practicing nurse: an integrative review of transition to practice programs. Nurse Educ Pract.

[15] Wray J, Watson R, Gibson H, Barrett D (2021). Approaches used to enhance transition and retention for newly qualified nurses (NQNs): a rapid evidence assessment. Nurse Educ Today.

[16] Shrestha S, Joshi S (2014). Lived experiences of the staff nurses during first six months of their employment in a university hospital. Kavre J Nepal Health Res Counc.

[17] Sandelowski M (2000). Whatever happened to qualitative description?. Res Nurs Health.

[18] Lincoln YS, Guba EG. Naturalistic inquiry. Sage; 1985.

[19] Creswell JW. Qualitative inquiry and research design: choosing among five approaches (2nd ed.). Sage. 2007.

[20] Tong A, Sainsbury P, Craig J (2007). Consolidated criteria for reporting qualitative research (COREQ): a 32-item checklist for interviews and focus groups. Int J Qual Health Care.

[21] Braun V, Clarke V (2006). Using thematic analysis in psychology. Qual Res Psychol.

[22] Younas A, Fàbregues S, Durante A, Escalante EL, Inayat S, Ali P (2023). Proposing the “MIRACLE” Narrative Framework for Providing Thick description in qualitative research. Int J Qualitative Methods.

[23] Tembo EN, Kabuluzi E, Gondwe W, Mbakaya BC (2019). Newly qualified registered nurses’ perceptions of the transition from student to qualified registered nurse: a qualitative study. Am J Nur & Prac.

[24] Fowler AC, Twigg D, Jacob E, Nattabi B (2018). An integrative review of rural and remote nursing graduate programmes and experiences of nursing graduates. J Clin Nurs.

[25] Clark CM, Springer PJ (2012). Nurse residents’ first-hand accounts on transition to practice. Nurs Outlook.

[26] Missen K, McKenna L, Beauchamp A (2015). Work readiness of nursing graduates: current perspectives of graduate nurse program coordinators. Contemp Nurse.

[27] Oducado RM, Ubas-Sumagaysay NA (2019). New Graduate Nurses: transition difficulties, support needed and satisfaction with Work Environment. Support Needed and Satisfaction with Work Environment [Paper presentation].

[28] Hallaran AJ, Edge DS, Almost J, Tregunno D (2022). New nurses’ perceptions on transition to practice: a thematic analysis. Can J Nurs Res.

